# The analysis of low-dose glucocorticoid maintenance therapy in patients with primary nephrotic syndrome suffering from COVID-19

**DOI:** 10.3389/fmolb.2023.1326111

**Published:** 2024-01-11

**Authors:** Yong-Qi Li, Min Wu, Yu-Jia Wang, Yu-Xia Zhang, Jing Lu, Yi-Nan Zhao, Bo-Fan Ji, Zhi-Qing Chen, Ri-Ning Tang, Bi-Cheng Liu

**Affiliations:** ^1^ Institute of Nephrology, Zhongda Hospital, School of Medicine, Southeast University, Nanjing, China; ^2^ Institute of Nephrology, Zhongda Hospital, Nanjing Lishui People’ Hospital, Nanjing, China

**Keywords:** coronavirus disease 2019, nephrotic syndrome, low-dose glucocorticoids, symptoms, quality of life

## Abstract

**Objectives:** This study aimed to describe the effects of low-dose (prednisolone acetate 2.5–7.5 mg/day) glucocorticoids (GCs) maintenance therapy in patients with primary nephrotic syndrome (NS) suffering from coronavirus disease 2019 (COVID-19).

**Methods:** A single-center retrospective study of NS patients with COVID-19 infection in Zhongda Hospital Affiliated to Southeast University from 1 February 2022 to 31 March 2023 was conducted. All enrolled patients underwent renal biopsy for the pathological diagnosis and reached complete remission (CR) or near-CR before COVID-19 infection. According to the maintained therapy regimen, patients were divided into low-dose GCs group and non-GCs group.

**Results:** A total of 125 patients were enrolled in the study. Their median age was 46.0 ± 15.6 years, and the median value of 24-h urine protein was 0.77 g. The majority of these patients received treatment for more than 6 months, with a significant portion achieving CR (29.6%) or near-CR (43.2%). The leading cause of NS was membranous nephropathy (52%). There were no significant differences in the baseline characteristics between low-dose GCs and non-GCs group. As compared to those in the non-GCs group, patients receiving low-dose GCs treatment showed less fatigue or muscle weakness, smell disorder, palpitations, decreased appetite, taste disorder, dizziness, sore throat or difficult to swallow and fever (*p* < 0.05). Moreover, patients in the low-dose GCs group were with higher median quality of life scores (85.0) than in the non-GCs group (*p* = 0.001). Further serum inflammatory factor analysis indicated that interleukin-6 (IL-6) levels in the non-GCs group were significantly higher than that in the low-dose GCs group (*p* < 0.05).

**Conclusion:** Patients with NS in low-dose GCs maintenance therapy stage showed milder symptom, higher quality of life and decreased serum IL-6 levels compared to those, who were not on GCs maintenance therapy. These results suggest the beneficial effect of low-dose GCs therapy in NS patients with CR/near-CR suffering from COVID-19 infection.

## 1 Introduction

Coronavirus disease 2019 (COVID-19), caused by severe acute respiratory syndrome coronavirus-2 (SARS-CoV-2), has caused severe casualties and economic losses worldwide (Wiersinga et al., 2020). The typical clinical symptoms of COVID-19 include fever, sore throat, myalgia, fatigue, and poor appetite as well as potentially severe complications, including acute respiratory distress syndrome, arrhythmia, and shock (Huang et al., 2020). Previous studies have suggested that cytokine storms caused by overproduction of inflammatory factors may be involved in the pathogenesis of patients with COVID-19 disease. However, the use of glucocorticoids (GCs) in some specific patients such as glomerulonephritis (GN) and nephrotic syndrome (NS), for the treatment of COVID-19 is controversial. Several studies have demonstrated the alleviative and therapeutic effects of GCs on COVID-19 due to their role in inhibiting dysfunctional systemic inflammation (Hu et al., 2020), reducing inflammatory-coagulant-fibroproliferative effects (Villar et al., 2020), and rapidly decreasing C-reactive protein and interleukin-6 (IL-6) levels (Li et al., 2021). However, investigations found that GCs might prolong the viral replication time, delay viral clearance and increase the death risk (Hu et al., 2020; Bae et al., 2021; El-Saber Batiha et al., 2022) in patients with COVID-19 (Fujishima, 2020; Al-Kuraishy et al., 2021).

The primary NS was defined as a urinary protein level of ≥3.5 g/day and a serum albumin level of <3.0 g/dL. It is usually caused by primary glomerular diseases, such as membranous nephropathy, minimal change disease, and focal segmental glomerulosclerosis (Lai et al., 2022). With the development of lesions, different degrees of renal function impairment, such as hematuria, proteinuria, and elevated blood creatinine, while some patients eventually develop end-stage renal disease. GCs have the advantage of rapid anti-inflammatory effects and are widely used in NS (Ponticelli and Locatelli, 2018; Riedel et al., 2023). Studies have shown that GCs can effectively reduce proteinuria and delay renal decompensation in patients with NS (Schijvens et al., 2019). Immunosuppressive agents, including tacrolimus, mycophenolate mofetil and rituximab, are commonly administrated in NS patients. Tacrolimus is known as a fermentation product isolated from the genus *Streptomyces* (Chin et al., 2021). Mycophenolate mofetil is an organic compound and its active ingredient mycophenolic acid can inhibit the proliferation and differentiation of T lymphocytes and B lymphocytes (Karunamoorthy et al., 2020). Rituximab is a chimeric mouse/human monoclonal antibody that binds to CD20 on B lymphocytes and triggers an immune response to B cell lysis (Ravani et al., 2021). The usage of high-dose GCs combined with immunosuppressive agents is supposed to poses a significant risk for patients with COVID-19 complicated by NS (Albarrán-Sánchez et al., 2022). However, for the patients in remission with NS who are on GC maintenance therapy, there is currently a lack of research on the efficacy of low-dose GCs in patients with concomitant COVID-19.

In order to observe the impact of low-dose GCs in NS patients who were in remission after COVID-19 infection, a clinical investigation was conducted to compare the difference in the COVID-19 symptoms of patients undergoing with or without low-dose GCs therapy.

## 2 Methods

### 2.1 Subjects

**FIGURE 1:**
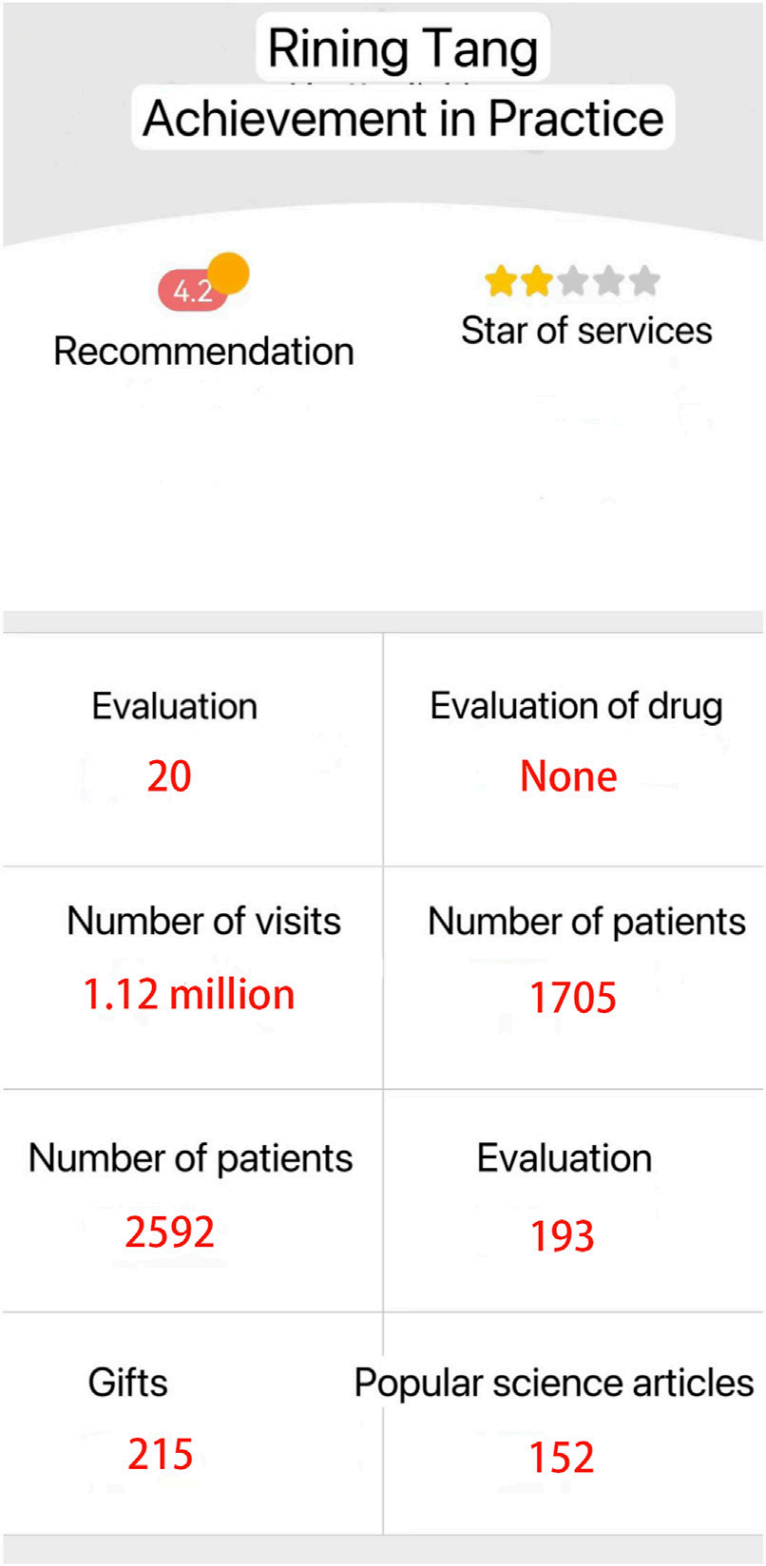
Online popular science platform.

A single-center retrospective observational study, including inpatients, who visited the Institute of Nephrology, Zhongda Hospital Affiliated with Southeast University from 1 February 2022, to 31 March 2023 was performed. Upon diagnosis, all NS patients received treatment with half-dose corticosteroids in combination with either tacrolimus, mycophenolate mofetil, or rituximab. After 3 months of treatment, all patients required oral sulfamethoxazole at a daily dose of 0.2–0.4 g. The tapering prednisolone acetate dose was reduced 20 mg/day and maintained for 1–2 months, followed by a reduction of 5 mg every 1–2 months until reaching a maintenance dose of 5 mg/day. During monthly follow-up appointments, there is a persistent focus on the prevention of upper respiratory infections and other precautions while on immunosuppressive therapy. Furthermore, online scientific educational materials are provided for patient awareness and education (Web address: https://www.haodf.com/doctor/2713404123.html) (Figure 1).

**FIGURE 2:**
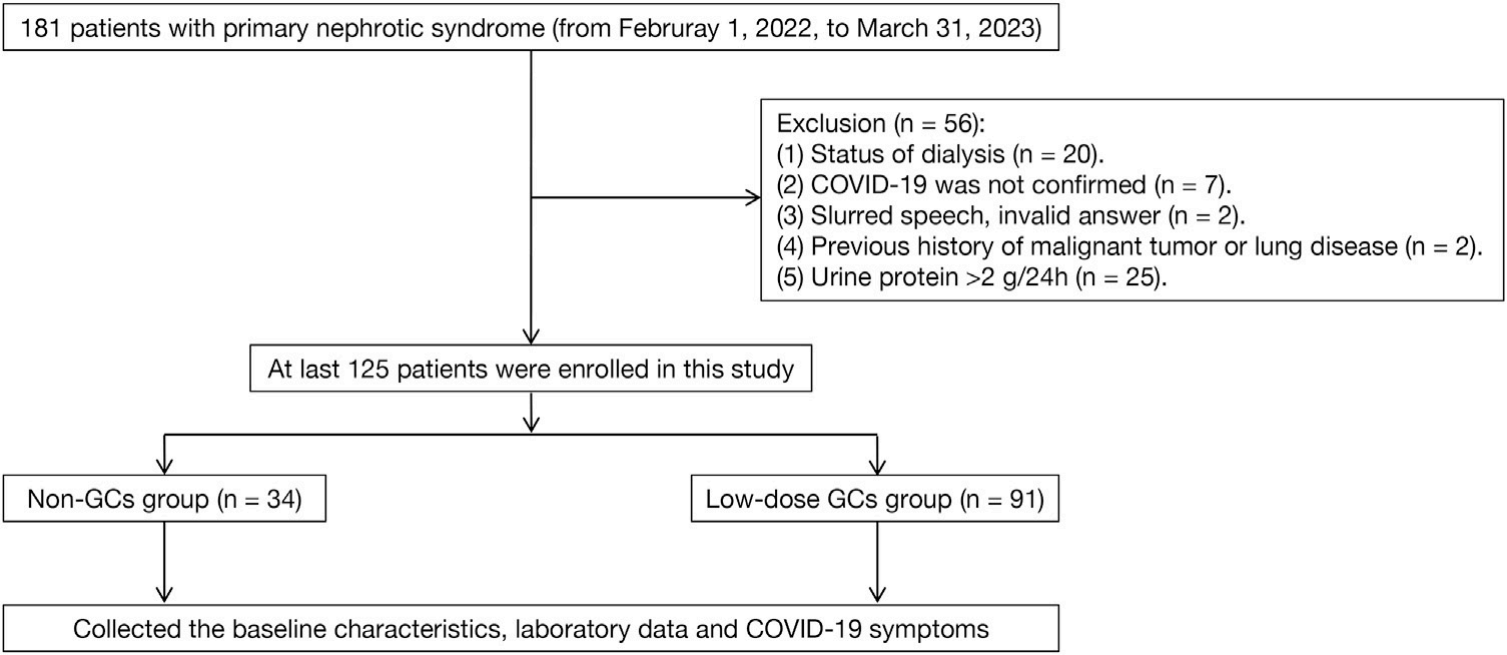
Flow chart of patients enrollment.

The inclusion criteria included: I) the patients with previous SARS-CoV-2 infection or current SARS-CoV-2 infection; II) previously diagnosed with NS and the specific pathological type was confirmed through renal biopsy; III) NS achieved favorable treatment outcomes in cases of CR (defined as urine protein level ≤0.3 g/24 h) or near-CR (defined as more than 80% reduction in proteinuria). The exclusion criteria included: I) the patients with a previous history of malignancy, pulmonary disease, or systemic disease that may involve the lungs; II) the patients, who had entered dialysis or kidney transplantation; and III) the patients with dementia or mental disorders that prevented them regular communication; IV) diagnosed with secondary NS. The screening process for the study subjects is shown in Figure 2.

The recruited patients were divided into low-dose GCs group and non-GCs group based on whether they were taking oral GCs (prednisolone acetate) during SARS-CoV-2 infection.

### 2.2 Data collection

The clinical data of patients, including gender, age, history of chronic diseases, blood pressure, education, smoking history, body mass index (BMI), and coronavirus vaccination history, were retrieved from electronic medical records at the time of COVID-19 infection. Cardiovascular diseases refer to acute or chronic heart failure, myocardial infarction, coronary artery disease, congenital heart disease, cardiomyopathy, etc. Cerebrovascular diseases include cerebral infarction, cerebral hemorrhage, cerebral aneurysm, cerebrovascular atherosclerosis, etc. The SARS-Cov-2 vaccine is an inactivated vaccine.

Baseline characteristics related to renal diseases included estimated glomerular filtration rate (eGFR), 24-h urine protein, and pathological types of glomerulonephritis. The COVID-19-related clinical symptoms included fatigue or muscle weakness, sleep difficulties, hair loss, smell disorder, palpitations, joint pain, decreased appetite, taste disorder, dizziness, diarrhea or vomiting, chest pain, sore throat or challenging to swallow, skin rash, myalgia, headache, and fever. The modified British Medical Research Council (mMRC) dyspnea scale, a five-category scale that correlates higher scores with more dyspnea, was used to describe the degree of dyspnea during physical exercise (Mahler and Wells, 1988). The EuroQol five-dimension five-level (EQ-5D-5L) questionnaire was used to measure the patient’s quality of life by assessing their mobility, self-care, regular activities, pain or discomfort, and anxiety or depression (Herdman et al., 2011). A patient’s subjective evaluation of their general health was performed by measuring the EuroQol Visual Analogue Scale (EQ-VAS), which ranged from 0 to 100; higher scores indicated a better subjective health experience (Rabin and de Charro, 2001).

Four researchers collected the patients’ data by querying the hospital medical record system and using a telephonic survey and were blind to GC status. The laboratory data, including white blood cell (WBC), red blood cell (RBC), hemoglobin (Hb), percentage of neutral cells (NEUT%), IL-6, interleukin-8 (IL-8), and C-reaction protein high sensitivity (CRP-HS) of patients, who tested positive for SARS-CoV-2, were collected. The study was approved by the Research Ethics Commission of Zhongda Hospital Affiliated to Southeast University (ethics No. 2023ZDSYLL144-P01), and the study was granted waiver of documentation of informed consent.

### 2.3 Statistical analyses

Categorical variables were expressed as frequency ratios and percentages, and continuous variables were expressed as mean, median, and interquartile range (IQR) values. Independent group t-tests were performed to compare means of continuous variables for the normally distributed data, while Mann-Whitney U-tests were used for the non-normally distributed data. The proportions of categorical variables were compared using the chi-square tests; however, Fisher’s exact test was used when the data were limited. All the statistical analyses were performed using SPSS (Statistical Package for Social Sciences) version 25.0 software. For the unadjusted comparisons, a two-sided *p*-value of <0.05 was considered statistically significant.

## 3 Results

### 3.1 General characteristics of the enrolled patients

As shown in Figure 2, a total of 125 patients were enrolled in this study. The demographic and clinical characteristics of the enrolled patients are listed in Table 1. The median age was 46.0 ± 15.6 years. The most common comorbidity was hypertension (*n* = 67, 53.6%), followed by type 2 diabetes (*n* = 17, 13.6%), cardiovascular diseases (*n* = 13, 10.4%), and cerebrovascular diseases (*n* = 8, 6.4%). Besides, 15 (44.1%) and 39 (42.9%) patients in the non-GCs and low-dose GCs group received COVID-19 vaccination, respectively. There were no significant differences in the baseline characteristics between the low-dose GCs and non-GCs groups.

**TABLE 1:** Characteristics of enrolled patients.

	Total (*n* = 125)	Non-GCs group (*n* = 34)	Low-dose GCs group (*n* = 91)	*p*-value
Age, years	46.0 *±* 15.6	43.4 *±* 14.9	47.0 *±* 15.9	0.246
Sex				0.427
Men	55 (44.0%)	13 (38.2%)	42 (46.2%)	
Women	70 (56.0%)	21 (61.8%)	49 (53.8%)	
Comorbidities				
Hypertension	67 (53.6%)	15 (44.1%)	52 (57.1%)	0.194
Diabetes	17 (13.6%)	4 (11.8%)	13 (14.3%)	0.942
Cardiovascular diseases	13 (10.4%)	5 (14.7%)	8 (8.8%)	0.526
Cerebrovascular diseases	8 (6.4%)	1 (2.9%)	7 (7.7%)	0.579
Systolic blood pressure ≥140 mmHg	41 (32.8%)	10 (29.4%)	31 (34.1%)	0.622
Diastolic blood pressure ≥90 mmHg	28 (22.4%)	9 (26.5%)	19 (20.9%)	0.505
Education				0.892
High school or lower	93 (74.4%)	25 (73.5%)	68 (74.7%)	
College or higher	32 (25.6%)	9 (26.5%)	23 (25.3%)	
Cigarette smoking				0.478
Never-smoker	97 (77.6%)	24 (70.6%)	73 (80.2%)	
Current smoker	7 (5.6%)	3 (8.8%)	4 (4.4%)	
Former smoker	21 (16.8%)	7 (20.6%)	14 (15.4%)	
BMI, kg/m^2^	23.0 *±* 3.2	23.7 *±* 3.7	22.8 *±* 2.9	0.183
Coronavirus vaccination history	54 (43.2%)	15 (44.1%)	39 (42.9%)	0.899

GCs, glucocorticoids; BMI, body mass index.

All the patients included in this study underwent renal biopsy. The proportions of the pathological types are listed in Table 2. Among them, membranous nephropathy had the highest percentage (*n* = 65, 52.0%), followed by mesangial proliferative glomerulonephritis (*n* = 35, 28.0%) and glomerular podocyte lesions (*n* = 19, 15.2%). A total of 65 patients (52.0%) had eGFR <90 mL/min per 1·73 m^2^, and the median value of 24-h urine protein for all the patients was 0.77 g.

**TABLE 2:** Basic information in patients with low-dose GCs and non-GCs group.

	Total (*n* = 125)	Non-GCs group (*n* = 34)	Low-dose GCs group (*n* = 91)	*p*-value
eGFR <90 mL/min per 1·73 m^2^	65 (52.0%)	19 (55.9%)	46 (50.5%)	0.595
24-h urine protein, g	0.77 (0.56)	0.56 (0.75)	0.80 (0.99)	0.078
Pathological types				0.013*
Glomerular podocyte lesions	19 (15.2%)	4 (11.8%)	15 (16.5%)	
Minimal change disease	4 (3.2%)	0 (0.0%)	4 (4%)	
Focal segmental glomerulosclerosis	7 (5.6%)	1 (2.9%)	6 (6.6%)	
Other	3 (2.4%)	1 (2.9%)	2 (2.2%)	
Mesangial proliferative glomerulonephritis	35 (28.0%)	16 (47.1%)	19 (20.9%)	
IgA nephropathy	35 (28.0%)	18 (52.9%)	17 (18.7%)	
Non-IgA nephropathy	7 (5.6%)	1 (2.9%)	6 (6.6%)	
Membranoproliferative glomerulonephritis	6 (4.8%)	0 (0.0%)	6 (6.6%)	
Membranous nephropathy	65 (52.0%)	14 (41.2%)	51 (56.0%)	
Dose of prednisone acetate, mg/day	7.5 (5.0)	NA	7.5 (5.0)	NA

GCs, glucocorticoids; eGFR, estimated glomerular filtration rate; *p < 0.05.

### 3.2 Analysis of COVID-19-related symptoms and laboratory data of enrolled patients

The proportion of included patients with any of the COVID-19-related symptoms was significant higher in the non-GCs group as compared to that in the low-dose GCs group (85.3% vs. 62.6%, *p* = 0.003) (Table 3). Among the 16 items of COVID-19-related symptoms listed in this study, the most common symptom was fever (*n* = 69, 55.2%), followed by fatigue or muscle weakness (*n* = 65, 52.0%), sore throat or difficulty swallowing (*n* = 40, 32.0%) and decreased appetite (*n* = 35, 28.0%), while the less frequent symptoms included hair loss (*n* = 2, 1.6%), chest pain (n = 4, 3.2%), and skin rash (*n* = 5, 4.0%).

**TABLE 3:** Symptoms of COVID-19, exercise capacity, and health-related quality of life at follow-up.

	Total (*n* = 125)	Non-GCs group (*n* = 34)	Low-dose GCs group (*n* = 91)	*p*-value
Symptoms of COVID-19				
Any one of the following symptoms	86 (68.8%)	29 (85.3%)	57 (62.6%)	0.003*
Fatigue or muscle weakness	65 (52.0%)	27 (79.4%)	38 (41.8%)	<0.001*
Sleep difficulties	14 (11.2%)	6 (17.6%)	8 (8.8%)	0.281
Hair loss	2 (1.6%)	0 (0.0%)	2 (2.2%)	0.944
Smell disorder	12 (9.6%)	8 (23.5%)	4 (4.4%)	0.004*
Palpitations	17 (13.6%)	9 (26.5%)	8 (8.8%)	0.023*
Joint pain	15 (12.0%)	4 (11.8%)	11 (12.1%)	1.000
Decreased appetite	35 (28.0%)	17 (50.0%)	18 (19.8%)	0.001*
Taste disorder	20 (16.0%)	13 (38.2%)	7 (7.7%)	<0.001*
Dizziness	25 (20.0%)	16 (47.1%)	9 (9.9%)	<0.001*
Diarrhoea or vomiting	9 (7.2%)	2 (5.9%)	7 (7.7%)	1.000
Chest pain	4 (3.2%)	1 (2.9%)	3 (3.3%)	1.000
Sore throat or difficult to swallow	40 (32.0%)	18 (52.9%)	22 (24.2%)	0.002*
Skin rash	5 (4.0%)	2 (5.9%)	3 (3.3%)	0.886
Myalgia	20 (16.0%)	7 (20.6%)	13 (14.3%)	0.392
Headache	21 (16.8%)	8 (23.5%)	13 (14.3%)	0.219
Fever	69 (55.2%)	27 (79.4%)	42 (46.2%)	0.001*
mMRC score				0.599
0	92 (73.6%)	24 (70.6%)	68 (74.7%)	
1	17 (13.6%)	6 (17.6%)	11 (12.1%)	
2	14 (11.2%)	4 (11.8%)	10 (11.0%)	
3	2 (1.6%)	0 (0.0%)	2 (2.2%)	
4	0 (0.0%)	0 (0.0%)	0 (0.0%)	
EQ-5D-5L questionnaire				
Mobility: problems with walking around	22 (17.6%)	9 (26.5%)	13 (14.3%)	0.111
Personal care: problems with washing or dishing	12 (9.6%)	7 (20.6%)	5 (5.5%)	0.027*
Usual activity: problems with usual activity	19 (15.2%)	8 (23.5%)	11 (12.1%)	0.113
Pain or discomfort	44 (35.2%)	16 (47.1%)	28 (30.8%)	0.090
Anxiety or depression	13 (10.4%)	4 (11.8%)	9 (9.9%)	1.000
Quality of life	80.0 (20.0)	70.0 (22.5)	85.0 (30.0)	0.001*

GCs, glucocorticoids; mMRC, score, modified British Medical Research Council; EQ-5D-5L questionnaire, EuroQol five-dimension five-level; *p < 0.05.

Notably, the rate of symptoms was higher in the non-GCs group as compared to those in the low-dose GCs group, showing significant differences in fever, fatigue or muscle weakness, sore throat or dysphagia, decreased appetite, smell disorder, palpitations, taste disturbance, and dizziness. The mMRC dyspnea scale showed no significant differences in the degree of dyspnea between the two groups. The EQ-5D-5L questionnaire showed that patients in the low-dose GCs group had fewer problems in personal care (5.5% vs. 20.6%, *p* = 0.027) as compared to those of the patients in the non-GCs group. The median quality of life score of all the patients was 80.0. And patients in the low-dose GCs group were with higher median quality of life scores (85.0) than that in the non-GCs group (70.0) (*p* = 0.001).

Regarding the test results, we collected the laboratory data of 39 inpatients and performed a comparative analysis. The results showed that RBC, Hb, and NEUT% were significantly higher, while the IL-6 level was significantly lower in the low-dose GCs group as compared to those in the non-GCs group (*p* < 0.05). However, there were no significant differences in the levels of WBC and CRP-HS (Table 4).

**TABLE 4:** Differences in laboratory results between the non-GCs and low-dose GCs group.

	Total (*n* = 39)	Non-GCs group (*n* = 15)	Low-dose GCs group (*n* = 24)	*p*-value
WBC, 10^9/L	8.5 ± 4.0	8.0 ± 4.7	8.9 ± 3.6	0.303
RBC, 10^9/L	3.9 ± 0.7	3.5 ± 0.5	4.2 ± 0.6	0.001*
Hb, g/L	115.3 ± 21.7	101.1 ± 19.0	124.7 ± 18.4	0.001*
NEUT%, %	70.6 ± 13.4	68.6 ± 14.5	72.0 ± 12.9	0.046*
IL-6, pg/mL	2.5 (4.5)	4.4 (7.0)	2.5 (3.7)	0.043*
IL-8, pg/mL	7.0 ± 6.5	10.0 ± 9.0	5.0 ± 3.0	0.108
CRP-HS, mg/L	3.6 (5.0)	0.9 (1.8)	0.8 (7.1)	0.764

GCs, glucocorticoids; WBC, white blood cell; RBC, red blood cell; Hb, hemoglobin; NEUT%, percentage of neutral cells; IL-6, interleukin-6; IL-8, interleukin-8; CRP-HS, C-reaction protein high sensitivity; *p < 0.05.

### 3.3 Subgroup analysis

In order to further analyze the effects of different pathological types on COVID-19 symptoms in low-dose GCs group, the included patients were divided into four groups: glomerular podocyte lesions, mesangial proliferative glomerulonephritis, membranoproliferative glomerulonephritis, membranous nephropathy. There were no significant differences in the symptoms among the four groups (*p* > 0.05) (Supplementary Table S1).

Furthermore, the effects of vaccination had an impact on COVID-19 symptoms were compared. The result showed no significant difference in the low-dose GCs group (*p* > 0.05) (Supplementary Table S2).

To explore whether the presence of type 2 diabetes has an impact on the manifestation of COVID-19 symptoms in the low-dose GCs group, a comparative analysis of COVID-19 symptoms between patients with and without type 2 diabetes was conducted. The results showed no statistically significant differences were observed between the two groups (*p* > 0.05) (Supplementary Table S3).

As shown in Supplementary Table S4, 91 patients using low-dose GCs were further divided into two groups: GCs-only group and GCs combined with immunosuppressant group. And the result showed on significant differences on symptoms of COVID-19, the degree of dyspnea and quality of life between these two groups.

## 4 Discussion

In this retrospective observational study of 125 patients, the COVID-19 symptomatology characteristics were summarized in the patients with NS. The data suggested that the patients with CR or near-CR receiving low-dose GCs maintenance therapy had milder COVID-19 symptoms, higher quality of life, and lower IL-6 levels as compared to those who did not receive GCs therapy. The subgroup analysis indicated that in the low-dose GCs group, there were no significant differences in COVID-19 symptoms based on renal pathological types, history of COVID-19 vaccination, and history of type 2 diabetes.

It is widely recognized COVID-19 has caused widespread global concern since its first report in December 2019, resulting in massive human infections and economic disruption. The use of GCs remains a significant controversy, mainly its timing and dosage having no valid definitive conclusion yet (Fujishima, 2020). However, the ability of GCs to treat severe COVID-19 patients has been demonstrated that GCs treatment could reduce all-cause mortality in a prospective meta-analysis including 7 randomized clinical trials and 1703 critically ill COVID-19 patients from different countries on five continents (Chaudhuri et al., 2021). The results of 3 randomized clinical trials (RCTs) (Angus et al., 2020; Dequin et al., 2020; Tomazini et al., 2020) showed that the use of dexamethasone could improve the short-term survival of COVID-19 patients requiring respiratory assistance. In addition, data from pandemic studies in China have provided compelling evidence, suggesting that methylprednisolone treatment was beneficial for patients with ARDS (Wu et al., 2020).

Meanwhile, the Chinese guidelines for the diagnosis and treatment of COVID-19 recommend the use of GCs for patients with severe COVID-19 (Diagnosis, 2023). However, non-severe patients, such as those with a history of NS, complete or partial remission of proteinuria, who were previously considered a high-risk group for COVID-19. Moreover, the recommendation and dosage of GC therapy for these patients are still unknown. Interestingly, low-dose GCs maintenance therapy could actually ameliorate the exacerbation of COVID-19 infection while also mitigating their COVID-19 symptoms in patients with GN. Thus, our study provides a valuable supplementary contribution to current guidelines and consensus.

In fact, GCs are the most widely used class of drugs worldwide, and their efficacy in the treatment of acute or chronic inflammatory conditions is undisputed (Vandewalle et al., 2018; Escoter-Torres et al., 2019). GCs inhibit the body’s inflammatory storm and reduce the damage caused by the inflammatory response to the pathogen (Reichardt et al., 2021; Ye et al., 2021). IL-6, an important pro-inflammatory factor (Potere et al., 2021), is associated with high SARS-CoV-2-induced inflammatory responses as well as a predictor of disease severity in COVID-19 patients (Han et al., 2020). Its higher level indicates a stronger inflammatory response and greater damage to the organism. In this study, the level of a key indicator reflecting COVID-19 inflammation, serum IL-6 levels, were notably reduced in patients undergoing low-dose GCs therapy for NS as compared to those in the control group. This observation accounted for the milder clinical symptoms observed in the GCs group.

It is worth mentioning that there were no statistical differences in the essential characteristics of the patients except for the pathological types of GN according to our data. For all patients with NS included in the study, the initial treatment regimen was all half-dose GCs (0.5 mg/kg/day) combined with tacrolimus, mycophenolate mofetil, or rituximab according to the above clinical integrated management. After at least 6 months of treatment, the median maintenance dose of prednisolone acetate used for the patients included in this study was 7.5 mg/day. Therefore, a very high CR or near-CR (72.8%) rate in NS patients was acquired within 1 year, which was much higher than the remission rate previously reported (Jha et al., 2007; Ruggenenti et al., 2012). Patients presented with remarkably mild COVID-19 symptoms, indicating a potential therapeutic effect of lower-dose GCs in patients with concurrent NS and SARS-CoV-2 infection. Nevertheless, a study evaluating the effects of early, low-dose, short-term GCs therapy on patients with non-severe COVID-19 showed prolonged fever duration and viral clearance as well as severe disease in the GCs group (Li et al., 2020). The differences in results might be due to differences in the timing of GCs administration, dosage, and participants’ characteristics. And it was worth mentioning that patients in the low-dose GCs group did not receive additional increased GC dosage during COVID-19.

The active exploration of GCs for a virus- or bacteria-induced inflammatory diseases is warranted because the application of steroid hormones for both SARS and other disease epidemics are in debate. Medications are also very carefully advised for patients with NS due to the risk for renal dysfunction, immune dysfunction, especially in elderly patients. Therefore, there is a need to accumulate clinical evidence on the use of GCs for in this disease. The current study attempted to supplement this gap, although providing limited data.

There were limitations to the current study. First, this was a single-center observational study. Second, as the laboratory data was derived from only a subset of patients, it might not be representative of the entire COVID-19-infected patient population, potentially resulting in limitations in the study’s findings. Third, there is a lack of subgroup analyses for SARS-CoV-2 mutants due to the lack of clinical data on SARS-CoV-2 mutants.

## Data Availability

The raw data supporting the conclusion of this article will be made available by the authors, without undue reservation.
