# Supplementary material for: The analysis of low-dose glucocorticoid maintenance therapy in patients with primary nephrotic syndrome suffering from COVID-19
Source: Front Mol Biosci. 2024 Jan 11;10:1326111. doi: 10.3389/fmolb.2023.1326111 (PMC10808412; doi:10.3389/fmolb.2023.1326111)
Supplement: Supplementary file 1 [file DataSheet1.docx]

**Supplementary Table S1:** Comparison of COVID-19 symptoms among different pathological types

in low-dose GCs group.

|  | **Glomerular podocyte lesions**  **(n = 15)** | **Mesangial proliferative glomerulonephritis (n = 19)** | | **Membranoproliferative glomerulonephritis (n = 6)** | **Membranous**  **nephropathy**  **(n = 51)** | | **P value** |
| --- | --- | --- | --- | --- | --- | --- | --- |
| **Symptoms of COVID-19** |  | |  |  |  |  | |
| **Any one of the following symptoms** | 8(53.3%) | | 11(57.9%) | 4(66.7%) | 34(66.7%) | 0.772 | |
| **Fatigue or muscle weakness** | 6(40.0%） | | 7(36.8%) | 3(50.0%) | 22(43.1%) | 0.937 | |
| **Sleep difficulties** | 1(6.7%) | | 1(5.3%) | 1(16.7%) | 5(9.8%) | 0.832 | |
| **Hair loss** | 0(0.0%) | | 0(0.0%) | 0(0.0%) | 2(3.9%) | 0.503 | |
| **Smell disorder** | 1(0.0%) | | 0(0.0%) | 0(0.0%) | 4(7.8%) | 0.189 | |
| **Palpitations** | 1(6.7%) | | 1(5.3%) | 1(16.7%) | 5(9.8%) | 0.832 | |
| **Joint pain** | 2(13.3%) | | 3(15.8%) | 0(0.0%) | 6(11.8%) | 0.615 | |
| **Decreased appetite** | 3(20.0%) | | 5(26.3%) | 2(33.3%) | 8(15.7%) | 0.647 | |
| **Taste disorder** | 0(0.0%) | | 1(5.3%) | 0(0.0%) | 6(11.8%) | 0.206 | |
| **Dizziness** | 0(0.0%) | | 1(5.3%) | 0(0.0%) | 8(15.7%) | 0.087 | |
| **Diarrhoea or vomiting** | 1(6.7%) | | 3(15.8%) | 1(16.7%) | 2(3.9%) | 0.369 | |
| **Chest pain** | 1(6.7%) | | 1(5.3%) | 1(16.7%) | 0(0.0%) | 0.123 | |
| **Sore throat or difficult to swallow** | 5(33.3%) | | 3(15.8%) | 2(33.3%) | 12(23.5%) | 0.636 | |
| **Skin rash** | 1(6.7%) | | 1(5.3%) | 0(0.0%) | 1(2.0%) | 0.718 | |
| **Myalgia** | 2(13.3%) | | 2(10.5%) | 2(33.3%) | 7(13.7%) | 0.650 | |
| **Headache** | 1(6.7%) | | 3(15.8%) | 1(16.7%) | 8(15.7%) | 0.801 | |
| **Fever** | 6(40.0%) | | 9(47.4%) | 3(50.0%) | 24(47.1%) | 0.961 | |
| **mMRC score** |  | |  |  |  | 0.519 | |
| **0** | 12(80.0%) | | 14(73.7%) | 4(66.7%) | 38(74.5%) |  | |
| **1** | 1(6.7%) | | 4(21.1%) | 1(16.7%) | 5(9.8%) |  | |
| **2** | 3(13.3%) | | 1(5.3%) | 0(0.0%) | 7(13.7%) |  | |
| **3** | 0(0.0%) | | 0(0.0%) | 1(16.7%) | 1(2.0%) |  | |
| **4** | 0(0.0%) | | 0(0.0%) | 0(0.0%) | 0(0.0%) |  | |
| **EQ-5D-5L questionnaire** |  | |  |  |  |  | |
| **Mobility: problems with walking around** | 2(13.3%) | | 1(5.3%) | 3(50.0%) | 7(13.7%) | 0.116 | |
| **Personal care: problems with washing or dishing** | 2(13.3%) | | 1(5.3%) | 1(16.7%) | 1(2.0%) | 0.276 | |
| **Usual activity: problems with usual activity** | 2(13.3%) | | 1(5.3%) | 1(16.7%) | 7(13.7%) | 0.734 | |
| **Pain or discomfort** | 6(40.0%) | | 7(36.8%) | 1(16.7%) | 14(27.5%) | 0.618 | |
| **Anxiety or depression** | 2(13.3%) | | 1(5.3%) | 1(16.7%) | 5(9.8%) | 0.805 | |
| **Quality of life** | 90.0(20.0) | | 80.0(30.0) | 80.0(33.8) | 80.0(20.0) | 0.255 | |

*GCs, glucocorticoids; mMRC score, modified British Medical Research Council; EQ-5D-5L questionnaire, EuroQol five-dimension five-level.*

**Supplementary Table S2:** Comparison of COVID-19 symptoms in low-dose GCs group based on COVID-19 vaccination status.

|  | **Coronavirus vaccination history** | | **P-value** | |
| --- | --- | --- | --- | --- |
|  | **No (n=52)** | **Yes (n=39)** | |  |
| **Symptoms of COVID-19** |  |  | |  |
| **Any one of the following  symptoms** | 30(57.7%) | 27(69.2%) | | 0.260 |
| **Fatigue or muscle weakness** | 21(40.4%) | 17(43.6%) | | 0.759 |
| **Sleep difficulties** | 6(11.5%) | 2(5.1%) | | 0.487 |
| **Hair loss** | 2(3.8%) | 0(0.0%) | | 0.606 |
| **Smell disorder** | 0(0.0%) | 4(10.3%) | | 0.065 |
| **Palpitations** | 5(9.6%) | 3(7.7%) | | 1.000 |
| **Joint pain** | 5(9.6%) | 6(15.4%) | | 0.610 |
| **Decreased appetite** | 9(17.3%) | 9(23.1%) | | 0.494 |
| **Taste disorder** | 2(3.8%) | 5(12.8%) | | 0.233 |
| **Dizziness** | 6(11.5%) | 3(7.7%) | | 0.800 |
| **Diarrhoea or vomiting** | 3(5.8%) | 4(10.3%) | | 0.691 |
| **Chest pain** | 3(5.8%) | 0(0.0%) | | 0.351 |
| **Sore throat or difficult to  swallow** | 12(23.1%) | 10(25.6%) | | 0.777 |
| **Skin rash** | 3(5.8%) | 0(0.0%) | | 0.351 |
| **Myalgia** | 8(15.4%) | 5(12.8%) | | 0.729 |
| **Headache** | 7(13.5%) | 6(15.4%) | | 0.795 |
| **Fever** | 23(44.2%) | 19(48.7%) | | 0.671 |
| **mMRC score** |  |  | | 0.504 |
| **0** | 38(73.1%) | 30(76.9%) | |  |
| **1** | 6(11.5%) | 5(12.8%) | |  |
| **2** | 6(11.5%) | 4(10.3%) | |  |
| **3** | 2(3.8%) | 0(0.0%) | |  |
| **4** | 0(0.0%) | 0(0.0%) | |  |
| **EQ-5D-5L questionnaire** |  |  | |  |
| **Mobility: problems with walking around** | 7(13.5%) | 6(15.4%) | | 0.795 |
| **Personal care: problems with washing or dishing** | 3(5.8%) | 2(5.1%) | | 1.000 |
| **Usual activity: problems with usual activity** | 7(13.5%) | 4(10.3%) | | 0.643 |
| **Pain or discomfort** | 13(25.0%) | 15(38.5%) | | 0.169 |
| **Anxiety or depression** | 7(13.5%) | 2(5.1%) | | 0.336 |
| **Quality of life** | 80.0(30.0) | 85.0(20.0) | | 0.845 |

*GCs, glucocorticoids; mMRC score, modified British Medical Research Council; EQ-5D-5L questionnaire, EuroQol five-dimension five-level.*

**Supplementary Table S3:** Comparing COVID-19 symptoms in low-dose GCs group between patients with and without type 2 diabetes

|  | **Type 2 diabetes history** | | | **P-value** | | |
| --- | --- | --- | --- | --- | --- | --- |
|  | **No (n=78)** | **Yes (n=13)** | |  | | |
| **Symptoms of COVID-19** |  | |  | |  |  |
| **Any one of the following symptoms** | 48(61.5%) | | 9(69.2%) | | 0.825 |  |
| **Fatigue or muscle weakness** | 31(39.7%) | | 7(53.8%) | | 0.340 |  |
| **Sleep difficulties** | 6(7.7%) | | 2(15.4%) | | 0.706 |  |
| **Hair loss** | 1(1.3%) | | 1(7.7%) | | 0.661 |  |
| **Smell disorder** | 4(5.1%) | | 0(0.0%) | | 0.917 |  |
| **Palpitations** | 8(10.3%) | | 0(0.0%) | | 0.496 |  |
| **Joint pain** | 10(12.8%) | | 1(7.7%) | | 0.948 |  |
| **Decreased appetite** | 15(19.2%) | | 3(23.1%) | | 1.000 |  |
| **Taste disorder** | 5(6.4%) | | 2(15.4%) | | 0.574 |  |
| **Dizziness** | 8(10.3%) | | 1(7.7%) | | 1.000 |  |
| **Diarrhoea or vomiting** | 7(9.0%) | | 0(0.0%) | | 0.574 |  |
| **Chest pain** | 2(2.6%) | | 1(7.7%) | | 0.905 |  |
| **Sore throat or difficult to swallow** | 21(26.9%) | | 1(7.7%) | | 0.250 |  |
| **Skin rash** | 2(2.6%) | | 1(7.7%) | | 0.905 |  |
| **Myalgia** | 12(15.4%) | | 1(7.7%) | | 0.760 |  |
| **Headache** | 11(14.1%) | | 2(15.4%) | | 1.000 |  |
| **Fever** | 37(47.4%) | | 5(38.5%) | | 0.548 |  |
| **mMRC score** |  | |  | | 0.090 |  |
| **0** | 59(75.6%) | | 9(69.2%) | |  |  |
| **1** | 11(14.1%) | | 0(0.0%) | |  |  |
| **2** | 7(9.0%) | | 3(23.1%) | |  |  |
| **3** | 1(1.3%) | | 1(7.7%) | |  |  |
| **4** | 0(0.0%) | | 0(0.0%) | |  |  |
| **EQ-5D-5L questionnaire** |  | |  | |  |  |
| **Mobility: problems with walking around** | 9(11.5%) | | 4(30.8%) | | 0.160 |  |
| **Personal care: problems with washing or dishing** | 4(5.1%) | | 1(7.7%) | | 1.000 |  |
| **Usual activity: problems with usual activity** | 8(10.3%) | | 3(23.1%) | | 0.393 |  |
| **Pain or discomfort** | 23(29.5%) | | 5(38.5%) | | 0.746 |  |
| **Anxiety or depression** | 7(9.0%) | | 2(15.4%) | | 0.830 |  |
| **Quality of life** | 85.0(30.0) | | 80.0(20.0) | | 0.163 |  |

*GCs, glucocorticoids; mMRC score, modified British Medical Research Council; EQ-5D-5L questionnaire, EuroQol five-dimension five-level.*

**Supplementary Table S4.** Comparison of COVID-19 symptoms between the two groups：GCs-only and GCs combined with immunosuppressant group.

|  | **Total**  **(n = 91)** | **GCs-only group**  **(n = 75)** | **GCs combined with immunosuppressant group**  **(n = 16)** | ***P* value** |
| --- | --- | --- | --- | --- |
| **Symptoms of COVID-19** |  |  |  |  |
| **Any one of the following symptoms** | 57(62.6%) | 46(61.3%) | 11(68.8%) | 0.578 |
| **Fatigue or muscle weakness** | 38(41.8%) | 32(42.7%) | 6(37.5%) | 0.704 |
| **Sleep difficulties** | 8(8.8%) | 5(6.7%) | 3(18.8%) | 0.288 |
| **Hair loss** | 2(2.2%) | 1(1.3%) | 1(6.3%) | 0.781 |
| **Smell disorder** | 4(4.4%) | 2(2.7%) | 2(12.5%) | 0.285 |
| **Palpitations** | 8(8.8%) | 6(8.0%) | 2(12.5%) | 0.928 |
| **Joint pain** | 11(12.1%) | 10(13.3%) | 1(6.3%) | 0.714 |
| **Decreased appetite** | 18(19.8%) | 13(17.3%) | 5 (31.3%) | 0.205 |
| **Taste disorder** | 7(7.7%) | 5(6.7%) | 2(12.5%) | 0.781 |
| **Dizziness** | 9(9.9%) | 7(9.3%) | 2(12.5%) | 1.000 |
| **Diarrhoea or vomiting** | 7(7.7%) | 5(6.7%) | 2(12.5%) | 0.781 |
| **Chest pain** | 3(3.3%) | 3(4.0%) | 0(0%) | 0.966 |
| **Sore throat or difficult to swallow** | 22(24.2%) | 19(25.3%) | 3(18.8%) | 0.813 |
| **Skin rash** | 3(3.3%) | 3(4.0%) | 0(0%) | 0.966 |
| **Myalgia** | 13(14.3%) | 11(14.7%) | 2(12.5%) | 1.000 |
| **Headache** | 13(14.3%) | 11(14.7%) | 2(12.5%) | 1.000 |
| **Fever** | 42(46.2%) | 35(46.7%) | 7(43.8%) | 0.832 |
| **mMRC score** |  |  |  | 0.564 |
| **0** | 68(74.7%) | 56(74.7%) | 12(75.0%) |  |
| **1** | 11(12.1%) | 10(13.3%) | 1(6.3%) |  |
| **2** | 10(11.0%) | 8(10.7%) | 2(12.5%) |  |
| **3** | 2(2.2%) | 1(1.3%) | 1(6.3%) |  |
| **4** | 0(0%) | 0(0%) | 0(0%) |  |
| **EQ-5D-5L questionnaire** |  |  |  |  |
| **Mobility: problems with walking around** | 13(14.3%) | 12(16.0%) | 1(6.3%) | 0.536 |
| **Personal care: problems with washing or dishing** | 5(5.5%) | 4(5.3%) | 1(6.3%) | 1.000 |
| **Usual activity: problems with usual activity** | 11(12.1%) | 10(13.3%) | 1(6.3%) | 0.714 |
| **Pain or discomfort** | 28(30.8%) | 25(33.3%) | 3(18.8%) | 0.396 |
| **Anxiety or depression** | 9(9.9%) | 7(9.3%) | 2(12.5%) | 1.000 |
| **Quality of life** | 85.0(30.0) | 85.0(30.0) | 82.5(27.5) | 0.332 |

*GCs, glucocorticoids; mMRC score, modified British Medical Research Council; EQ-5D-5L questionnaire, EuroQol five-dimension five-level.*
